# Altered Neuronal Dynamics in the Striatum on the Behavior of Huntingtin Interacting Protein 14 (HIP14) Knockout Mice

**DOI:** 10.3390/brainsci3041588

**Published:** 2013-11-20

**Authors:** Ana María Estrada-Sánchez, Scott J. Barton, George V. Rebec

**Affiliations:** 1 Program in Neuroscience, Indiana University, Bloomington, IN 47405, USA; E-Mails: amestrad@indiana.edu (A.M.E.-S.); sjbarton@indiana.edu (S.J.B.); 2 Department of Psychological and Brain Sciences, Indiana University, Bloomington, IN 47405, USA

**Keywords:** Huntington’s disease, huntingtin interacting protein 14, striatum, local field potentials, motor inflexibility, plus maze, palmitoylation

## Abstract

Huntington’s disease (HD), a neurodegenerative disorder caused by an expanded CAG repeat in the huntingtin gene, impairs information processing in the striatum, which, as part of the basal ganglia, modulates motor output. Growing evidence suggests that huntingtin interacting protein 14 (HIP14) contributes to HD neuropathology. Here, we recorded local field potentials (LFPs) in the striatum as HIP14 knockout mice and wild-type controls freely navigated a plus-shaped maze. Upon entering the choice point of the maze, HIP14 knockouts tend to continue in a straight line, turning left or right significantly less often than wild-types, a sign of motor inflexibility that also occurs in HD mice. Striatal LFP activity anticipates this difference. In wild-types, the power spectral density pattern associated with entry into the choice point differs significantly from the pattern immediately before entry, especially at low frequencies (≤13 Hz), whereas HIP14 knockouts show no change in LFP activity as they enter the choice point. The lack of change in striatal activity may explain the turning deficit in the plus maze. Our results suggest that HIP14 plays a critical role in the aberrant behavioral modulation of striatal neuronal activity underlying motor inflexibility, including the motor signs of HD.

## 1. Introduction

Huntington’s disease (HD) is an inherited neurodegenerative disorder in which behavioral signs, mainly characterized by involuntary movements and cognitive decline, typically emerge in adulthood [1]. Ample evidence indicates that impaired communication between cortical and striatal networks is a leading indicator of the HD behavioral phenotype [2,3]. Neurons in striatum are especially vulnerable to mutant huntingtin (mHTT), the protein underlying HD [4,5]. Among the changes induced by mHTT is a deficit in protein palmitoylation by huntingtin interacting protein 14 (HIP14), a palmitoyl acyl transferase (PAT) that binds palmitic acid [6,7,8]. Failure to palmitoylate key proteins can impair synaptic transmission, which may account for the altered response of striatal neurons to cortical activation in HD mouse models [9]. Consistent with a role for HIP14 in HD, mice lacking HIP14 develop striatal neuropathology and motor deficits similar to those described for transgenic HD models [10].

When transgenic HD mice are allowed to explore a plus-shaped maze, they are less likely than wild-type controls to turn into the left or right arm of the maze and continue moving instead into the opposite arm, a sign of motor inflexibility that also occurs in HD patients [11,12,13]. We reported similar behavior in HIP14 knockouts, which was accompanied by abnormal firing patterns in individually isolated striatal neurons [14]. At the choice point or center of the maze, these neurons, relative to those recorded from wild-type mice, change both the rate and pattern of spike activity. Firing rate increases as does the number of spikes that cluster together in bursts. Collectively, these results, which implicate HIP14 in striatal function, suggest that aberrant striatal processing may underlie the motor inflexibility seen in HIP14 knockout mice. Here, we extended this investigation to the population response of striatal neurons by analyzing local field potentials (LFPs) in conjunction with entering and exiting the choice point. A force-plate actometer monitored position in the maze and provided time-stamps for LFP activity.

## 2. Results and Discussion

**Figure 1:**
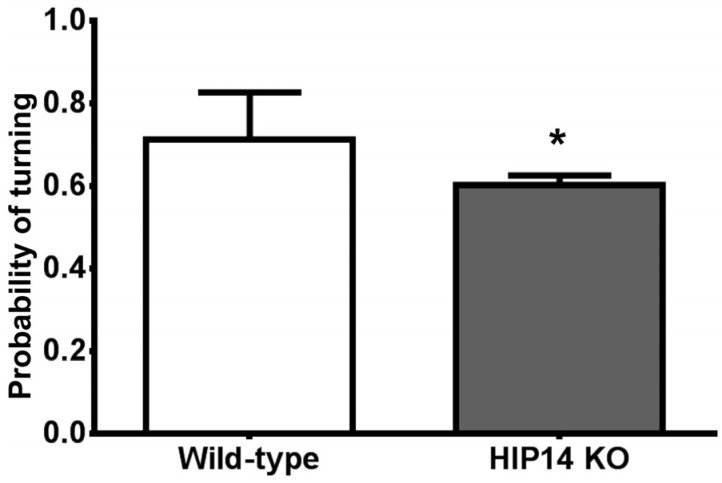
HIP14 knockouts (HIP14 KO) are less likely to turn left or right as they explore the plus-shaped maze. The graph shows the turning probability obtained after wild-type and HIP14 knockout mice freely explored the plus maze for 30 min. The probability was obtained from the sum of arm choices to the right or left arm divided by the total number of arm choices. Wild-type and HIP14 knockout mice participated in multiple sessions for a total of 38 and 46 sessions, respectively. Data were analyzed by the unpaired *t*-test. Data are expressed as the mean ± SEM. *t* = 3.6; df = 82; * *p* = 0.0001.

When mice enter the center or choice point of the plus maze, they have the option of continuing straight into the opposite arm or turning into either the left or right arm [11]. Wild-type (*n* = 10) and HIP14 knockout mice (*n* = 10) participated in multiple recording sessions; each mouse participated in at least two sessions for a total of 38 and 46 sessions, respectively, for each group. As Figure 1 shows, wild-type mice turn to explore the perpendicular arms with a probability of 0.71 ± 0.01. In contrast, HIP14 knockout mice are less likely to turn (the probability of turning is 0.60 ± 0.02); instead, once they cross the choice point, they explore the opposite arm. Statistical analysis of the probability of turning revealed a significant difference between wild-type and HIP14 knockout mice (*t* = 3.6, degrees of freedom (df) = 82, *p* = 0.0005). 

The reduced probability of turning in HIP14 knockout mice is not due to the fact that they were less active than wild-type. In fact, relative to wild-type, HIP14 knockouts were more active when exploring the plus maze, as evidenced by the total number of arm choices observed: wild-type = 69.11 ± 4.6; HIP14 knockouts = 150.9 ± 11.37 (*t* = 6.19, df = 82; *p* < 0.0001). Thus, although HIP14 knockouts are more active in the plus maze than wild-types, the HIP14 knockouts turn less, a common sign of motor inflexibility [12].

**Figure 2:**
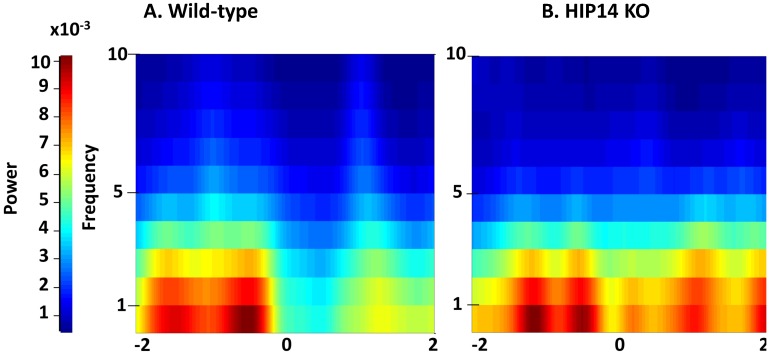
Striatal local field potential (LFP) activity recorded from wild-type and HIP14 knockout mice before and after entry into the choice point. Spectrogram plots were obtained 2 s before and 2 s after the wild-type (**A**) and HIP14 knockout (**B**) mice enter the center of the maze (denoted by zero). Graphs were obtained from the average of the total of crossing events obtained from 5 wild-type (339 events) and 6 HIP14 knockout mice (701 events).

Because evaluating patterns of arm choice provides information on behavioral flexibility, we focused our analysis of striatal LFP activity on events surrounding the choice point. We first constructed spectrograms based on total entries into the choice point obtained from five randomly selected wild-type (339 entries) and six HIP14 knockout mice (701 entries). As shown in Figure 2, two seconds before entering the choice point (denoted by zero), both groups showed high power at low frequencies [delta (0.1–4 Hz), theta (4–7 Hz)]. Note, however, that when the mice cross the choice point, LFP power decreases only in wild-types; HIP14 knockouts maintain the same level of power at low frequencies.

**Figure 3:**
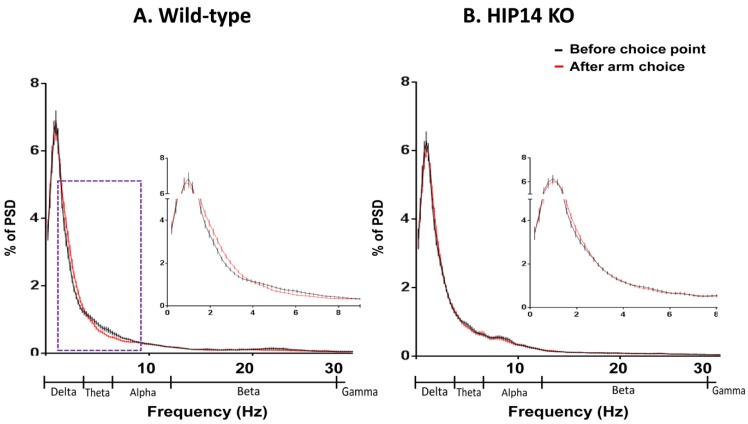
Mean power spectral density (PSD) plotted as a proportion of total power (% PSD) for wild-type (**A**) and HIP14 knockout (**B**) mice immediately before choice-point entry (*black*) and after arm entry (*red*). For comparison purposes, 50 random events of a one-second duration for each recording session from each mouse (43 sessions for wild-types and 44 for HIP14 knockouts) were measured. The dashed box denotes the frequencies at which the mean PSDs significantly differ. Insets provide a close up of activity at frequencies <8 Hz. PSD data were obtained from 50 random events per recording session. Data are expressed as the mean ± SEM (*p* ≤ 0.05).

Next, we compared choice-related LFP activity with LFP activity when the animal had subsequently moved into an arm. For this comparison, we analyzed the second immediately before choice-point entry with the second after the mouse entered an arm. In order to make the LFP comparison comparable between groups, since HIP14 knockout mice crossed the center of the maze more often than wild-type animals, we analyzed a total of 50 randomly selected choice and arm events from each mouse (regardless of the direction of the turn). Mean power spectral density (PSD) data are shown in Figure 3. LFP activity in the arm was significantly decreased relative to choice-point activity in wild-type mice (*p* ≤ 0.05), at delta (0.1–4 Hz), while theta (4–7 Hz) bands activity increased (Figure 3A). Whereas HIP14 knockouts showed no change in power as they moved from choice point to arm (Figure 3B). Thus, protein palmitoylation by HIP14 might be involved in behavioral flexibility in arm choice and for the differential responsiveness of striatal LFP activity to position in the plus maze.

HIP14 knockout mice are less likely to turn left or right when they navigate the plus maze (Figure 1). Instead, they typically continue moving, straight into the opposite arm; and, when they eventually make a left or right turn, they again persist in exploring the opposite arm. This behavior is a sign of motor inflexibility, which refers to a decreased likelihood of alternating arm entries [12]. Given that the striatum constitutes part of the neuronal circuitry that shapes motor control, our data suggest that a change in low frequency striatal LFP activity plays a critical role in shaping behavioral choice.

**Figure 4:**
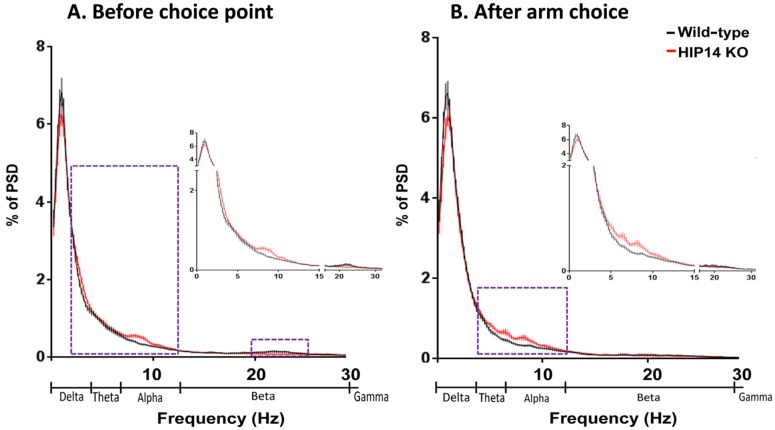
Striatal PSD immediately before the choice point (**A**) and after arm entry (**B**) for wild-type (*black*) and HIP14 knockout mice (*red*). In each case, data are based on one second of LFP activity obtained from 50 random events per recording session (43 wild-type and 44 HIP14-knockout sessions). Dashed boxes denote the frequencies at which the mean PSD significantly differ. Data are expressed as the mean ± SEM (*p* ≤ 0.05). Insert: close up of frequencies at which differences were observed.

As shown in Figure 4, we also compared choice-related and arm-related LFP activity between wild-type and HIP14 knockout mice. Striatal LFPs in HIP14 knockout mice relative to wild-type show significant increases in delta (0.1–4 Hz) and alpha (8–13 Hz) frequencies and decreases in beta (13–30 Hz) immediately before the choice point, but increases in the theta (4–7 Hz) and alpha (8–13 Hz) range after arm entry.

Overall, our analysis indicates that when HIP14 knockout mice navigate the plus maze, striatal LFPs maintain an increase in power at lower frequencies, both before and after the choice point, which may contribute to behavioral inflexibility. A summary of averaged striatal LFPs at relevant frequencies is provided in Table 1 for both wild-type and HIP14 knockout mice.

**Table 1:** Average of % of power spectral density in wild-type and HIP14 knockout mice.

Frequency	Wild-type		HIP14 KO	
	Before	After	Before	After
Delta (0.1–4 Hz)	143.7 ± 0.08 *	152.8 ± 0.07	149.1 ± 0.07	151.1 ± 0.07
Theta (4–7 Hz)	33.4 ± 0.04 *	27.8 ± 0.03 ^†^	35.9 ± 0.03	35.0 ± 0.03
Alpha (8–13 Hz)	10.8 ± 0.01 *^,†^	13.3 ± 0.01 ^†^	14.5 ± 0.02 *	18.4 ± 0.02
Beta (13–30 Hz)	4.1 ± 0.01	3.4 ± 0.009	3.4 ± 0.008	3.3 ± 0.006
Gamma (30–50 Hz)	1.0 ± 0.003	0.9 ± 0.003	0.9 ± 0.002	0.9 ± 0.002

* Statistically different relative to neuronal activity obtained while mice explore the arm *p* < 0.05; ^†^ Relative to neuronal activity observed in HIP14 knockout *p* < 0.05. Data were obtained from the average of % PSD (Figure 3 and Figure 4) from each frequency. Data were analyzed by two-way ANOVA; data expressed as mean ± SEM. *n* = 43 recording sessions for wild-type and *n* = 44 for HIP14 knockout.

Our results suggest that alerted striatal dynamics in HIP14 knockouts may underlie the behavioral signs of motor inflexibility observed in arm-choice selection in the plus maze. Along with altered LFP, we previously reported that striatal neurons in HIP14 knockout mice show an increased firing rate and reduced synchronized activity at the choice point of the maze [14]. HIP14 knockouts also show deficits in nest-building behavior, a test that provides information on motor coordination and cognitive function [10,14,15]. Thus, HIP14 function, which includes protein palmitoylation, plays a critical role in striatal processing and appears necessary for normal motor output. More studies are necessary to understand the potential mechanisms (e.g., kynurenine metabolites interacting with the *N*-methyl-d-aspartate NMDA receptor) underlying impaired medium spiny neuron (MSN) processing and altered neuronal dynamics in mice lacking the HIP14 protein.

## 3. Experimental Section

### 3.1. Animal Housing and Genotype

Animal use followed the National Institutes of Health (Bethesda, MD, USA) guidelines and was approved by the Institutional Animal Care and Use Committee (Bloomington, IN, USA). HIP14 knockout and wild-type littermates (FVB/N background strain) were bred from heterozygous pairs obtained from the Hayden Laboratory at the University of British Columbia, Vancouver, Canada. Mice were housed individually and maintained under controlled temperature and humidity conditions with a 12-h light/dark cycle and food and water *ad libitum*. Genotyping was carried out as previously described [14].

### 3.2. Electrode Implantation Surgery and LFP Recordings

Ten HIP14 knockout mice (age 30–35 weeks, mean age 33.16 ± 0.6 weeks) and 10 wild-type littermate controls (age 30–35, mean age 32.3 ± 0.4 weeks) underwent electrode surgery implantation. Each electrode bundle consisted of four 50 μm Formvar-insulated stainless steel wires (California Fine Wire, Grover Beach, CA, USA) and one 50 μm uninsulated stainless steel ground wire. Two bundles were friction-fitted to gold pin connectors in a custom nylon hub (6-mm diameter); the electrode assembly is small, lightweight and well tolerated by the mice, so that they could behave freely. Mice were anesthetized with an intraperitoneal injection of a mixture of chloral hydrate and sodium pentobarbital (170 mg/kg chloral hydrate, 40 mg/kg sodium pentobarbital; 0.4 mL/100 g body weight) and mounted in a stereotaxic frame. Following a midline scalp incision, a hole was drilled through the skull to target the striatum (+0.8 mm anterior and ±2.2 mm lateral to bregma and 3.2 mm ventral) [14]. Two additional holes were drilled in the contralateral site for stainless anchor screws. Electrodes were lowered into the striatum, and the electrode assembly was then permanently attached to the skull by means of dental acrylic.

After 1 week of postsurgical recovery, electrode assembly was connected to a lightweight flexible wire harness equipped with field-effect transistors that provided unity-gain current amplification for each of the eight microwires. Then, the mouse was placed in our transparent plus-shaped maze made of Plexiglas^®^ (Arkema Inc., Bristol, PA, USA) (arm 25 cm long × 5 cm wide with the side 30 cm high) [12]. The plus maze was suspended 2 mm above a force-plate actometer, a device that monitors the position of the mouse and indicates the number of turns into each arm (right, left, front and back) and also indicates when the mouse crossed over the choice point by placing a timestamp on the LFP data [16]. LFP, routed through preamplifiers with 1000× gain and 0.7–170 Hz filters, were sampled at 1000 Hz and acquired by a multichannel acquisition processor (Plexon, Dallas, TX, USA). Recording sessions were conducted once weekly for 30 min each when the mice were between 30 and 52 weeks of age. A total of *n* = 43 recording sessions for wild-type and *n* = 44 for HIP14 knockout were obtained.

### 3.3. Data Analysis

LFP data for each recording session was analyzed by means of NeuroExplorer (Nex Technologies, Littleton, MA, USA).

### 3.4. Statistical Analysis

Statistic program package GraphPad Prism 6 (GraphPad Software, San Diego, CA, USA) was used for statistical analysis. Spectrograms (Figure 1A,B) were obtained from the average LFP activity in five wild-type and six HIP14 knockouts; the data were graphed in MATLAB (MATrix LABoratory; The MathWorks, Natick, MA, USA). Plus maze turning data were analyzed by the unpaired *t*-test. Each frequency in mean PSD curves was compared by a *t*-test. Two-way ANOVA was used to compare striatal LFP frequencies when the mice were at the choice point *versus* in the plus-maze arm. Data is expressed as the mean ± SEM; differences were considered significant when *p* ≤ 0.05.

## 4. Conclusions

Striatal LFPs oscillations in wild-type mice show changes at lower frequencies (<10 Hz) as they approach to the center and choose the next arm to explore. On the contrary, HIP14 knockout mice lack changes at any of the LFPs frequencies; instead, similar striatal LFP activity is observed before mice approach the choice point and after they have chosen the arm they will explore. Interestingly, HIP14 knockout mice are less likely to make a 90-degree turn when they explore the plus maze. Thus, altered striatal LFPs modulation might underlie motor inflexibility in HIP14 knockout mice.
